# Round the Hospitals

**Published:** 1921-09-24

**Authors:** 


### ROUND THE HOSPITALS.

The need for hostels for women is urgent. Such is the pronouncement of the sub-committee of the Housing Advisory Council, and it is made after very close investigation into the circumstances. Among all the working women who have difficulty in finding suitable quarters in which to pursue their avocations nurses are in the worst position. Their irregular hours and the exhausting nature of their duties render some degree of home comfort indispensable if their health is not to suffer. In the ordinary boarding-house they are often deprived of the possibility of day-time sleep, and they have a strong claim to be specially considered in regard to the accommodation provided for them. The committee recommends the provision of both large and small hostels in various localities, with cubicles at 7s. to 9s. a week according to locality, and self-contained rooms at from 12s. to 20s. per week. The recommendations, even if not immediately adopted, are a definite step in the right direction.

We seem to be very far yet from protecting the name of "nurse" from being misappropriated by impostors. A woman who described herself as a nurse, apparently on no foundation whatever, was recently sent to prison for fourteen days with hard labour, for being drunk and disorderly. When asked by the chairman at the Croydon Borough Police Court, where she was charged, why she described herself as a nurse, she declared she had been applying for situations as a nurse. That her name should have been entered on the charge-sheet as a nurse does undoubtedly, as Dr. Fowler plainly told her, bring discredit on the profession. This is a kind of discredit which registration can do little to remedy. Can the police be instructed to consider none but the registered genuine nurses? We fear not.

The new matron of the Herefordshire General Hospital is Miss Constance Keys-Wells, lately sister-housekeeper at the Royal Sussex County Hospital, Brighton. She is to be congratulated on securing the appointment out of no fewer than 150 applicants, three of whom "all excellent" were finally selected for the Committee to interview. Miss Keys-Wells had long-continued training, first with children at the Royal Hospital for Sick Children, Edinburgh, and then at the Charing Cross Hospital. From 1916 to 1920 she was doing war work, etc. It is a fortunate circumstance that the new matron is a trained sick children's nurse, for the county hospitals are apt to overlook this important branch of specialism, and we have more than once been struck by the dull quarters assigned in such establishments to the little ones and the lack of interest displayed in their surroundings and welfare.

The Hayward's Heath Cottage Hospital was recently inspected by Miss Lloyd Still, R.R.C., C.B.E., and we understand that the matron, Miss Barrett, was much gratified by the expressions of approval which the arrangements called forth. It is to the cottage hospitals that many institutions unprovided with a preliminary training-school must look to take the place of this important feature in the training of young nurses. To bring the best of them into accord with the central scheme of training planned by the education committee of the General Nursing Council probably little is needed beyond a few technical alterations, and their position would be immeasurably strengthened if these were carried out. The qualifications of cottage hospital matrons are high, and the intimate relations into which they are thrown with their subordinate workers enables them to get a real grip over character and lay a valuable foundation.

The local centres of the College are entering on their autumn programme with zest, and many new developments are on foot. At Sheffield efforts are being made to organise a big jumble sale to take place on October 8 in the Lower Cutlers' Hall, in aid of the proposed nurses' club in the city, and all who have saleable articles of any kind, new or old, to spare are requested to communicate with the Hon. Treasurer, Miss C. Watson, at 426 Glossop Road, Sheffield. It is high time progress was made with this much-needed club and we hope to hear it is in being before the New Year.

THE presentation of medals to nurses who gain the highest marks in their final examination is a custom which is spreading and is found, generally speaking, for some matrons we know do not altogether believe in it, very successful in helping to keep up interest in every branch of the curriculum. The Belfast Union Infirmary, owing to the generosity of three guardians who have presented the medals, is now added to the list of institutions, which confer this honour on their nurses. At a recent meeting of the Board the prize-winners received their medals; Gold, Nurse Ireland; Silver, Nurse Kennedy; Bronze, Nurse McCartin. They were complimented by the Chairman on their success and by Miss Campbell, the Lady Superintendent, the competition for the first places having been particularly keen.

WORK on the new maternity home which is to be built at Hampstead from the sums remaining over from Queen Mary's Needlework Guild has now so far advanced that the foundation-stone will be laid by Her Majesty on Wednesday, October 12. The plans show accommodation for fifteen patients, who will be received from any part of the country and at varying rates. Pending the completion of permanent questions, the Home is being carried on at Cedar Lawn, Hampstead, which was opened in October 1919 and has proved up to the hilt the need for accommodation of this nature.

MR. DYER, the chairman of the Camberwell Infirmary Committee, recently entertained the entire nursing staff of that institution, with the matron, the medical superintendent, and several of the Guardians, at a garden-party in the grounds, which lasted from early afternoon till 10 P.M., so that all the nurses were able to be present during some part of the time. The local branch of the Y.M.C.A. lent their aid to organise sports and concerts with the most successful results. It was the first party of the kind which has ever taken place at the infirmary, and Mr. Dyer was much congratulated on his happy innovation.

A CURIOUS state of affairs has arisen at the Beverley Road Workhouse at Hull. Last June the Guardians appointed Sister Moore, who had been six and a-half years at the Sulcoates Infirmary, to be deputy superintendent nurse and home sister. The appointment was opposed by the medical officer and by the superintendent nurse, on the ground that the sister was not qualified for the post, and she was apparently not allowed to take up her duties. The Ministry of Health inspector looked into the matter, and advised the Board to reconsider the matter, and the House Committee, after long deliberation, urged the Board to ask Sister Moore to resign. Will it be believed that the Board have ignored the recommendations of the inspector and their own Committee, and have insisted on Sister Moore being given six months' trial? The discussion was seized upon as the occasion for an attack on the senior officers, and one more illustration of "how not to govern an institution" has been given to the ratepayers.

